# The association of the betatrophin level with metabolic and inflammatory parameters in infertile women with polycystic ovary syndrome: A case-control study

**DOI:** 10.18502/ijrm.v20i1.10406

**Published:** 2022-02-18

**Authors:** Fatemeh Keikha, Ensieh Shahrokh Tehraninejad, Marzieh Rakhshkhorshid, Malihe Afiat, Fedyeh Hagholahi, Fatemeh Ghasemi

**Affiliations:** ^1^Health Reproductive Research Center, Imam Khomeini Hospital, Tehran University of Medical Sciences, Tehran, Iran.; ^2^Department of Midwifery, School of Nursing and Midwifery, Mashhad University of Medical Sciences, Mashhad, Iran.; ^3^Milad Infertility Center, Department of Obstetrics and Gynecology, Faculty of Medicine, Mashhad University of Medical Sciences, Mashhad, Iran.

**Keywords:** ANGPTL8 protein human, Infertility, Polycystic ovary syndrome, Iran.

## Abstract

**Background:**

Betatrophin may be associated with metabolic diseases.

**Objective:**

To investigate the betatrophin level and its association with metabolic and inflammatory parameters in infertile women with polycystic ovary syndrome (PCOS) and other infertile women during the intrauterine insemination cycle.

**Materials and Methods:**

This case-control study was conducted with 90 infertile women (45 with PCOS and 45 without) chosen by convenience sampling, in the infertility clinic of Imam Khomeini Hospital in Tehran, Iran. Participants were interviewed to obtain their age, body mass index, and reproductive history. Fasting brachial venous blood samples were obtained on the 3 ${}^{\mathrm{rd}}$ day of the menstrual cycle to measure the levels of betatrophin, fasting blood sugar, insulin, luteinizing hormone, follicle-stimulating hormone, low-density lipoprotein cholesterol, estradiol, and high-sensitivity C-reactive protein.

**Results:**

The results showed that the level of betatrophin in women with PCOS was significantly higher than in the control group (p = 0.05). Based on multiple linear regression analyses, the effects of metabolic and inflammatory parameters on betatrophin were not significant (p = 0.19). The results showed no significant difference between groups in folliculogenesis (p = 0.57).

**Conclusion:**

According to the results, betatrophin levels were higher in infertile women with PCOS than in those without. The findings suggest that there may be an association between increased betatrophin and increased incidence of PCOS. Further studies with a larger sample size are needed to investigate the role of betatrophin in insulin resistance and lipid metabolism, and its effects on infertility treatment outcomes.

## 1. Introduction

Polycystic ovary syndrome (PCOS) is a common hormonal health problem that occurs in 5-10% of women of reproductive age (1). Ovulatory dysfunction and hyperandrogenism are two features of PCOS (2). It is hypothesized that the syndrome can be due to several metabolic disorders including low-grade chronic inflammation, resistance to insulin, metabolic syndrome, obesity, hyperlipidemia, glucose intolerance, hypertension, and/or an elevation of the risk of Type 2 diabetes (3).

Two of the most significant and highly prevalent parameters among women with PCOS are insulin resistance and metabolic disorders (4). As a consequence of resistance to insulin, betatrophin levels are expected to vary in the serum as well as in other biological compartments (5). Betatrophin is a peptide hormone derived from liver and adipose tissue (3). This protein regulates glucose homeostasis and lipid metabolism (6). An association between circulating betatrophin levels, Type 1 diabetes, and PCOS has been reported (5); however, the pathogenesis effects of betatrophin on the secretion of insulin and homeostasis of glucose are not fully known (2).

Recent clinical studies have confirmed a positive association between circulating betatrophin levels and serum lipid levels in participants with impaired glucose tolerance, participants with Type 2 diabetes, and healthy individuals. Furthermore, “it has been suggested that betatrophin may be associated with circulating low-density lipoprotein cholesterol (LDL-C), high-density lipoprotein cholesterol, and triglycerides (TG), probably via different mechanisms” (7). A study was conducted to find the relationship between betatrophin levels and anthropometric, hormonal, and metabolic parameters in women with PCOS with a body mass index (BMI) < 25 kg/m ${}^{2}$ who were glucose tolerant. The researchers studied 50 of such women using the Rotterdam criteria, and 60 healthy controls who were age and BMI matched and did not present any feature of clinical or biochemical hyperandrogenism. The study confirmed that serum betatrophin levels were significantly higher in glucose tolerant women with PCOS who had a BMI < 25 kg/m ${}^{2}$ than in the control group (8).

The results of another study with the aim of investigating betatrophin serum levels in participants with newly-diagnosed PCOS and their influence demonstrated a positive correlation between the serum betatrophin levels in women with PCOS and their fasting blood glucose, TG, BMI, and waist-to-hip ratio (p = 0.01). However, serum betatrophin levels were uncorrelated with the fasting insulin and homeostasis model assessment of insulin resistance (HOMA-IR) (p = 0.05). Multiple stepwise regression analysis showed that the TG level was an independent influence of the serum betatrophin level. However, correlations mentioned above were not found in the control group (9). Many studies have examined the association between betatrophin and metabolic and inflammatory parameters and how they are linked to metabolic diseases (2, 3, 10).

In this study, we aimed to investigate the betatrophin level and its association with metabolic and inflammatory parameters in infertile women with and without PCOS during the intrauterine insemination (IUI) cycle.

## 2. Materials and Methods

This case-control study was conducted in the infertility clinic of Imam Khomeini Hospital in Tehran, Iran from September 2017 to November 2018.

45 infertile women with a diagnosis of PCOS and 45 infertile women with regular menstruation and with other causes of infertility (idiopathic or male factor) chosen by convenience sampling were included in the study. The participants entered the course of IUI. PCOS diagnosis was determined according to the Rotterdam PCOS Consensus criteria that require at least the presence of two of the following criteria: 1) ovulation dysfunction (anovulation or sporadic ovulation); 2) hyperandrogenism (clinical or biochemical); and 3) polycystic ovaries present on ultrasound (15 follicles 2-10 mm in diameter in either ovary) (11). The other inclusion criteria were: age of 20-40 yr; BMI < 35 kg/m^2^; at least one tubal patency; and normal levels of thyroid stimulating hormone and prolactin. Women with any underlying disease (e.g., diabetes, hypertension, cardiac or hepatorenal disease, chronic inflammatory disease, etc.), women who had had an infection in the previous two wk, women taking oral contraceptive pills, antiandrogens or other hormonal drugs in the last month, and women who use recreational drugs, smoke or drink alcohol were excluded.

Participants were interviewed to obtain age and reproductive history, including gravidity, parity, abortion, ectopic pregnancy, menstruation pattern, infertility duration, and stimulation duration. The height and weight of each participant were measured using a digital bathroom scale (Omron Company, Japan) and a SECA stadiometer (SECA Instruments Ltd, Germany). The measurements were recorded to the nearest 0.5 cm and 0.1 kg, respectively. BMI was calculated by dividing weight (in kilograms) by height squared (in meters) while the participants were weighed barefooted and in light clothing. Fasting brachial venous blood samples were taken to measure the levels of betatrophin, fasting blood sugar (FBS), insulin, luteinizing hormone (LH), follicle-stimulating hormone (FSH), LDL-C, estradiol, and high-sensitivity C-reactive protein (hs-CRP). The participants were on the 3 ${}^{\mathrm{rd}}$ day of their menstrual cycle at the time of the blood test. The collected blood was centrifuged at 4000 × g for 10 min right after the test and stored at -80°C until the analysis. To find the amounts of LH, FSH, hs-CRP, FBS, and estradiol, automated chemiluminescence immunoassay systems were used (BT 1500 Biotecnica instruments SPA, Italy). The Friedewald formula (12) and the enzyme-linked immunosorbent assay (ELISA) technique were used for the calculation of LDL-C and hs-CRP, respectively. The intra-assay coefficient of about 5% and inter-assay coefficient of about 10% were determined for each factor. To evaluate the HOMA index, fasting insulin and glucose were used. Insulin resistance was calculated by glucose (mmol/L) multiplied by fasting insulin (mU/L)/22.5 (HOMA-IR formula). The chemiluminescence immunometric assay and a commercial kit (Immulite 2000 Analyzer; CPC, USA) were used to measure fasting insulin. To measure fasting glucose, a glucose oxidase assay (Tosoh Corp., Tosoh, Japan) was used.

ELISA kits (EIAab Science, Wuhan, China; Catalogue No. E11644h) were used to assess fasting serum betatrophin levels following the manufacturer's instructions. The tests were performed in duplicate, and samples with more than 5% coefficient of variation were excluded.

Every participant in the case or control group was stimulated to ovulate with two clomiphene citrate tablets (Ovumid 50, Iran Hormone Pharmaceutical Co, Tehran, Iran) starting from the 3 ${}^{\mathrm{rd}}$ day of their menstrual cycle. On the 8 ${}^{\mathrm{th}}$ day of the cycle, 150 units of human menopausal gonadotropin (karma-HMG 75 u, Pharmatech GmbH, Germany) were administered. Following that, serial transvaginal sonography (Philips ultrasound, affinity 70, Japan) was performed until the dominant follicle reached a diameter of 18-20 mm. Subsequently, 10,000 units of human chorionic gonadotropin (karma-HCG 5000 u, Pharmatech GmbH, Germany) was prescribed and 36 hr later, participants were subjected to IUI. Two wk later, the level of beta-human chorionic gonadotropin was checked (MPR-404, ELISAMicroplate Reader, Roedermark, Germany) and, if positive, an ultrasound was performed to evaluate the pregnancy and observe the fetal heart rate.

### Ethical considerations

The study was approved by the Ethics Committee of the Tehran University of Medical Sciences (Code: IR.TUMS.VCR.REC.1396.2259). Written informed consent was obtained from all participants before the initiation of the study.

### Statistical analysis

The Statistical Package for the Social Sciences (SPSS, version 25, IBM Corp., Armonk, NY, USA) was used to analyse the data. The Shapiro-Wilk test was used to test the distribution of the data. Data were presented as mean ± SD or median (interquartile range). An independent *t* test and a Chi-square test were used to assess between-group differences for continuous variables and categorical variables, respectively. In order to compare group medians for data with non-normal distributions the Mann-Whitney U test was used. Spearman's Rank Correlation Coefficient was used to analyse bivariate relations between betatrophin levels and covariates. The determinants of betatrophin (independent variables: BMI, FSH, LH, LDL-C, hs-CRP, FBS, insulin, and HOMA-IR) were evaluated separately using multiple linear regression. Furthermore, the effect of betatrophin and HOMA-IR on pregnancy was investigated using logistic regression.

## 3. Results 

There were 45 participants in each group. Four participants from the control group withdrew from the study before its completion; they did not return after administration of clomiphene citrate for treatment. Age, reproductive history, FSH, LH, LDL-C, hs-CRP, FBS, and HOMA-IR were similar between the groups. As expected, women with PCOS had a higher mean BMI than the control group. Clinical and hormonal details of the participants in the case and control groups are shown in table I.

The results of the Chi-square test showed that 73.3% (33 people) of the case group had an irregular pattern of menstruation, while no participants in the control group had an irregular pattern of menstruation (p ≤ 0.001). Correlations between betatrophin concentration and clinical, metabolic, and inflammatory parameters of the participants are shown in table II.

Based on multiple linear regression analyses, the effects of BMI, FSH, LH, LDL-C, hs-CRP, FBS, insulin, and HOMA-IR on betatrophin were not significant (Table III). The results of the independent *t* test showed no significant difference between groups in folliculogenesis (p = 0.57). The results of the Chi-square test showed that gestational rates were 20.0% and 31.7% in the case and control groups, respectively; this difference was not statistically significant (p = 0.21). Based on the logistic regression model, betatrophin and HOMA-IR did not predict pregnancy.

**Table 1:** Clinical, metabolic and inflammatory parameters of participants in the case and control groups


** Variables **	**Cases**	**Controls**	**p-value**
** Age (yr) ${}^{\text{a}}$ **	29.75 ± 4.49	31.39 ± 5.34	0.12*
**BMI (kg/m^2^) ${}^{\text{a}}$ **	27.72 ± 5.14	23.34 ± 2.40	≤ 0.001*
**Gravidity ${}^{\text{b}}$ **	0 (0-2)	0 (0-4)	0.14**
**Parity ${}^{\text{b}}$ **	0 (0-2)	0 (0-1)	0.19**
**Abortion ${}^{\text{b}}$ **	0 (0-2)	0 (0-3)	0.44**
**EP ${}^{\text{b}}$ **	0 (0-1)	0 (0-0)	0.96**
**Infertility duration (yr) ${}^{\text{b}}$ **	4.0 (1.5-15.0)	3.0 (1.0-16.0)	0.08**
**Stimulation duration (cycle) ${}^{\text{b}}$ **	8 (4-14)	7 (2-14)	0.61**
**FSH (mIU/ml) ${}^{\text{a}}$ **	5.13 ± 1.87	5.64 ± 2.50	0.29*
**LH (mIU/ml) ${}^{\text{b}}$ **	6.70 (1.10-23.20)	5.30 (1.20-19.10)	0.19**
**LDL-C ${}^{\text{a}}$ **	111.28 ± 27.48	113.24 ± 22.82	0.72*
**hs-CRP ${}^{\text{b}}$ **	2 (1-32)	2 (0-7)	0.91**
**FBS (mg/dl) ${}^{\text{a}}$ **	91.72 ± 9.21	91.95 ± 7.22	0.90*
**Insulin (mIU/L) ${}^{\text{b}}$ **	19.50 (2.40-169.70)	12.60 (3.50-106.00)	0.03**
**Betatrophin ${}^{\text{b}}$ **	32.60 (16.70-218.80)	24.20 (18.50-216.50)	0.05**
**HOMA-IR ${}^{\text{b}}$ **	4.47 (0.58-39.39)	2.66 (0.78-22.77)	0.08**
${}^{\text{a}}$ Data presented as Mean ± SD, ${}^{\text{b}}$ Data presented as Median (Minimum-maximum). *Independent *t* test, **Mann-Whitney U-test. BMI: Body mass index, EP: Ectopic pregnancy, FSH: Follicle-stimulating hormone, LH: Luteinizing hormone, LDL-C: Low-density lipoprotein cholesterol, hs-CRP: High-sensitivity C-reactive protein, FBS: Fasting blood sugar, HOMA-IR: Homeostasis model assessment of insulin resistance			

**Table 2:** Spearman's rank correlation coefficients of betatrophin concentration and clinical, metabolic, and inflammatory parameters of participants


	**Cases**		**Controls**	
**Variables**	**r**	**p-value**	**r**	**p-value**
	**BMI**	0.010	0.94	-0.810	0.61
**FSH**	0.062	0.06	0.029	0.85
**LH**	0.179	0.23	0.041	0.07
**LDL-C**	-0.337	0.02	0.173	0.28
**hs-CRP**	-0.155	0.31	-0.155	0.33
**FBS**	-0.072	0.64	-0.141	0.38
**Insulin **	-0.047	0.76	-0.101	0.53
**HOMA-IR**	-0.071	0.64	-0.106	0.51
**Number of dominant follicles **	0.051	0.73	-0.218	0.17
Spearman's rank correlation coefficients. P-value ≤ 0.05 was considered significant (13), BMI: Body mass index, FSH: Follicle-stimulating hormone, LH: Luteinizing hormone, LDL-C: Low-density lipoprotein cholesterol, hs-CRP: High-sensitivity C-reactive protein, FBS: Fasting blood sugar, HOMA-IR: Homeostasis model assessment of insulin resistance				

**Table 3:** Results of multiple linear regression analysis for dependent variable betatrophin


** Variables**	**B**	β	**p-value**	**95% CI**
**BMI**	0.435	0.032	0.77	-2.583 - 3.452
**FSH**	3.056	0.109	0.35	-3.417 - 9.528
**LH**	2.780	0.215	0.05	-0.026 - 5.586
**LDL-C**	-0.559	-0.229	0.03	-1.083 - -0.036
**hs-CRP**	1.728	0.116	0.30	-1.583 - 5.039
**FBS**	-1.206	-0.161	0.28	-3.426 - 1.013
**Insulin**	-4.081	-2.084	0.26	-11.305 - 3.143
**HOMA-IR**	17.207	2.000	0.28	-14.513 - 48.927
Multiple linear regression analysis. P-value ≤ 0.05 was considered significant (13), BMI: Body mass index, FSH: Follicle-stimulating hormone, LH: Luteinizing hormone, LDL-C: Low-density lipoprotein cholesterol, hs-CRP: High-sensitivity C-reactive protein, FBS: Fasting blood sugar, HOMA-IR: Homeostasis model assessment of insulin resistance				

## 4. Discussion 

Our results showed that the level of betatrophin in women with PCOS was significantly higher than in the control group. In the present study assessing the association of metabolic parameters such as the lipid profiles with betatrophin levels, it was found that amongst lipid profiles only lower LDL-C levels were associated with betatrophin levels in women with PCOS. In addition, the results of the multiple linear regression analysis did not show any effect of any of the inflammatory or metabolic parameters on the betatrophin level.

Betatrophin is a peptide that is produced in the liver as well as in brown and white adipose tissue and it regulates the function of pancreatic beta cells and fat production (5). The results of studies on betatrophin vary. Some studies have shown an increase in betatrophin levels in women with PCOS (3, 14, 15). However, the results of one study showed that betatrophin levels were lower in women with PCOS than in the control group. They also found a significant negative correlation between betatrophin levels, and insulin, BMI and HOMA-IR. The results of their study showed that the only factor affecting beta-dopamine reduction was PCOS (2). Another study found that betatrophin concentration was lower in women with PCOS than in the control group. Their results showed that there was a positive correlation between betatrophin concentration and LDL-C in women with PCOS whose betatrophin concentrations were higher than the cut-off (464.5 ng/l) (6). The results of these studies are not in line with our study.

The results of a study on the association of betatrophin levels with PCOS showed a correlation between PCOS and fasting insulin levels as well as a significant correlation between the levels of betatrophin and fasting insulin levels and HOMA-IR in women with PCOS (14). A negative correlation between serum betatrophin levels and BMI, fasting insulin, and HOMA-IR was demonstrated in another study (15). The present study did not show such correlations. This difference in results may be due to differences in laboratory kits used, ages, ethnicities, or sample size.

In our study, women with PCOS had a higher mean BMI than the control group; but no significant correlation between betatrophin and BMI was found. The results of a recent study done on obese populations were not in line with this study (16). Also, some contradictory results regarding the association of betatrophin levels and obesity have been reported (17, 18). The results of a cross-sectional study showed that there was a significant positive correlation between betatrophin levels and BMI in women with PCOS (3), while the results of a case-control study showed the opposite (14). Another study aiming to investigate blood levels of betatrophin in obese children with non-alcoholic fatty liver disease reported no statistically significant difference between the control and experimental groups (19).

This study showed significantly higher levels of fasting insulin in women with PCOS compared to the control group. This indicated that the women with PCOS were insulin resistant. About 80% of women with PCOS have insulin resistance. Betatrophin is produced as a result of insulin resistance (14). The findings of some studies vary regarding the association of betatrophin levels with diabetes (17, 18, 20). In our study, we did not find a significant correlation between the levels of betatrophin and fasting insulin levels. One study showed a significant negative correlation between betatrophin levels and fasting insulin levels in women with PCOS (21), while another showed the opposite (14). The differences in race and lifestyle may have influenced the results of these studies.

Betatrophin has been linked to lipid metabolism changes. But studies' findings are conflicting. Betatrophin affects the lipid profile by regulating the secretion of very low-density lipoproteins from the liver and inhibiting lipoprotein lipase activity (22). Chinese researchers have reported that there is a positive correlation between betatrophin and TG (23). In another study, the results showed a negative correlation between circulating betatrophin levels and TG, but a positive correlation with high-density lipoprotein cholesterol (18). Furthermore, another study found no association between betatrophin levels and improved fertility outcomes in participants (3).

The results of the present study showed a significant negative correlation between the level of betatrophin and LDL-C in women with PCOS; therefore, it can be concluded that as the level of betatrophin increases, the level of LDL-C decreases. The results of this study were not in line with a cross-sectional study on PCOS women which showed a significant positive correlation between betatrophin and LDL-C levels (3). PCOS is a disease associated with mild chronic inflammation. However, in this study, there was no significant relationship found between betatrophin and inflammatory parameters such as hs-CRP.

One of the limitations of the present study was the small sample size and the attrition in the control group. Also, in the evaluation of insulin resistance, a non-invasive HOM-IR method that was less sensitive than rigorous testing of the euglycemic clamp was used. This may explain the lack of association found between betatrophin levels and insulin resistance in this study. Moreover, the subjects were not evaluated for betatrophin sequence variations.

## 5. Conclusion

According to the results, betatrophin levels were higher in infertile women with PCOS than in women with other causes of infertility. Given the findings, it can be concluded that there may be an association between increased betatrophin and increased incidence of PCOS. Further studies with a larger sample size are needed to investigate the relationship between betatrophin and insulin resistance and lipid metabolism, and its effects on infertility treatment outcomes.

##  Conflict of Interest

The authors declare that they have no conflict of interest.
